# Study on the Hot Deformation Behavior of Stainless Steel AISI 321

**DOI:** 10.3390/ma15124057

**Published:** 2022-06-07

**Authors:** Liudmila V. Radionova, Danil V. Perevozchikov, Aleksandr N. Makoveckii, Victor N. Eremin, Alexander M. Akhmedyanov, Sergey V. Rushchits

**Affiliations:** 1 Department of Metallurgy, Moscow Polytechnic University, Bolshaya Semyonovskaya Street 38, 107023 Moscow, Russia; 2 Chelyabinsk Pipe Rolling Plant, Mashinostroiteley Street 21, 454129 Chelyabinsk, Russia; danil.perevozchikov@chelpipegroup.com (D.V.P.); aleksandr.makoveckiy@chelpipegroup.com (A.N.M.); veremin@chelpipegroup.com (V.N.E.); 3 Department of Material Science and Physics and Chemistry of Materials, South Ural State University, Lenin Prospect 76, 454080 Chelyabinsk, Russia; akhmedianovam@susu.ru (A.M.A.); rushchitcsv@susu.ru (S.V.R.)

**Keywords:** hot deformation, dynamic recovery, dynamic recrystallization, Zener–Hollomon parameter, stainless steel, AISI 321

## Abstract

In this study, the hot deformation behavior of austenitic Ti-modified AISI 321 steel with a relatively high content of carbon (0.07 wt.%) and titanium (0.50 wt.%) was studied in the temperature range of 1000–1280 °C and strain rates in the range of 0.01–1 s^−1^. Hot deformation was carried out with uniaxial compression of cylindrical specimens on a Gleeble 3800 thermomechanical simulator. It is shown that the flow stress increased with a decrease in the deformation temperature and an increase in the strain rate. The shape of the stress-strain curves indicates that, at high temperatures and low strain rates, the hot deformation of AISI 321 steel was accompanied by dynamic recrystallization. The passage of dynamic recrystallization was confirmed by microstructural studies. Hyperbolic sine type of constitutive equation with deformation activation energy *Q* = 444.2 kJ·mol^−1^ was established by analyzing the experimental flow stresses. The power-law dependences of the critical strain necessary for the onset of dynamic recrystallization and the size of recrystallized grains on the Zener–Hollomon parameter were established. The value of the parameter *Z* = 5.6 × 10^15^ was determined, above which the dynamic recrystallization was abruptly suppressed in the steel under study. It is speculated that the suppression of dynamic recrystallization occurs due to dispersed precipitates of titanium carbonitrides.

## 1. Introduction

Austenitic stainless steels are widely used in the chemical industry, thermal and nuclear power, and medical and cryogenic engineering [1]. The production process of semi-finished products and final products from these steels usually includes the stage of hot deformation via rolling, forging, or extrusion [2,3,4].

Hot deformation of steels is accompanied by softening processes: dynamic recovery and recrystallization [5,6,7,8]. The mechanism of dynamic softening depends on temperature, strain and strain rate, grain size, stacking fault energy, and amount of precipitation [9,10,11]. Austenitic stainless steels have a low stacking fault energy [1]. This circumstance hinders the cross-slip and climb of dislocations, slows down the dynamic recovery processes, and contributes to the rapid accumulation of critical dislocation density necessary for the onset of dynamic recrystallization. The complete passage of dynamic recrystallization is capable of providing significant refinement of the initial coarse-grained structure during hot rolling [12,13].

The study of deformation behavior and structure evolution during hot deformation of austenitic stainless steels was mainly carried out on AISI 316 and AISI 304 steels [14,15,16,17,18]. In these and many other studies, analytical expressions have been obtained that predict, at a given temperature and strain rate, the value of flow stress, critical strain for initiation of dynamic recrystallization, and grain sizes.

In AISI 321 steels, titanium additives prevent the precipitation of chromium carbides and thereby reduce the tendency to intergranular corrosion [1,19,20]. Titanium additives also affect the behavior of AISI 321 steel during hot deformation. Thus, Nkhoma et al. [21] performed a comparative study of AISI 321 with 0.35% titanium and Ti-free AISI 304 steels and showed that the deformation activation energy of AISI 321 steel is higher than that of steel AISI 304. According to the authors [21], this difference is the result of the bounding of interstitial carbon and nitrogen atoms within coarse titanium carbonitrides that precipitate at high temperatures, as well as a higher content of δ-ferrite in AISI 321 steel. Differences in the microstructure of the two hot-deformed steels in [21] have not been studied. In a series of studies [22,23,24,25], Ghazani et al. performed a comprehensive study of the deformation behavior of AISI 321 steel with 0.04% carbon and 0.32% titanium in the temperature range of 850–1200 °C and strain rates of 0.001–1 s^−1^. Results showed that the main restoration process in the temperature range of 800–950 °C is dynamic recovery [22]. At a deformation temperature of 950 °C, partial dynamic recrystallization develops, and starting from a temperature of 1000 °C, the microstructure entirely consists of dynamically recrystallized grains [23]. In [24], the kinetics and critical conditions for the initiation of dynamic recrystallization were studied, and in [25], a method was proposed for predicting the flow curves of the steel under study as a function of temperature and strain rate. Haj et al. [26] investigated the hot deformation of AISI 321 steels with higher carbon (0.056%) and titanium (0.48%) content than those studied in [21,22,23,24,25]. In contrast to the results of [23], deformation at 1050 °C and a strain rate of 1 s^−1^ was accompanied by only partial dynamic recrystallization, and after deformation at a temperature of 950 °C and strain rate of 0.01 s^−1^, the microstructure consisted entirely of non-recrystallized grains with precipitated of complex carbides along the grain boundaries. It should be noted that the sample preheating temperature before subsequent deformation (1150 °C) was lower than those indicated in [21,22,23,24,25] (1200 °C). Therefore, a larger amount of undissolved titanium carbonitrides could remain in the steel before deformation. Kratochvil et al. [27] also studied AISI 321 steel with a high content of carbon (0.06%) and titanium (0.52%). The samples were directly heated to deformation temperatures in the range of 850–1180 °C (without preliminary high-temperature annealing). Therefore, as the authors admit, it is difficult to interpret the temperature dependence of the flow stress due to the large and different number of precipitates for each deformation temperature. In the same study [27], hot rolling of seamless pipes was simulated by two successive compression deformations at temperatures of 1050 °C and 950 °C. Two-stage deformation was not accompanied by dynamic recrystallization, and subsequent annealing at 1050 °C did not cause significant static recrystallization, although a completely recrystallized structure was formed in the steel with a lower content of carbon and titanium after similar treatment. According to the authors of [27], the reason for the delay in static recrystallization during annealing of steel with a high content of carbon and titanium is the dispersed particles of titanium carbonitrides precipitated during cooling after hot deformation. Indeed, such particles have been detected by transmission electron microscopy. However, their results [27] do not exclude the possibility of retaining some of the carbonitrides particles present in the initial state or their precipitation during hot deformation. Thus, the features of hot deformation of steel with a higher content of carbon and titanium require further research.

The aim of the present study was to investigate the processes of hot deformation of AISI 321 steel containing 0.07% carbon and 0.5% titanium in the range of temperatures and strain rates typical for rolling operations in the production of seamless pipes. For the most complete dissolution of titanium carbonitrides, the preheating temperature of the samples (1280 °C) was chosen to be equal to the heating temperature of the billet in the production process.

## 2. Materials and Methods

The chemical composition of the austenitic stainless steel used in the present study is shown in Table 1.

The samples in the form of cylinders with 15 mm length and 10 mm diameter were cut out of the tube after piercing, in the radial direction. Hot deformation was carried out using a uniaxial compression test on a simulator of thermomechanical processes, Gleeble 3800. The temperature of the sample during the test was measured using a thermocouple welded to its central part. Before deformation, the samples were heated to a temperature of 1280 °C and held for 10 min. Notably, 1280 °C is a typical temperature for heating cast blanks before subsequent hot deformation. Afterward, the sample was cooled to the deformation temperature, held at this temperature for 3 min, and deformed. The deformation was carried out at temperatures of 1000 °C, 1100 °C, 1200 °C, and 1280 °C with a strain rate of 0.01 s^−1^, 0.1 s^−1^, and 1 s^−1^. During the test, the true strain ε, the true stress σ, and the current temperature of the sample *T* were recorded.

To study the structure formed during hot deformation, samples were quenched via a water jet after the deformation was completed. Microstructural studies were performed using an optical microscope C. Zeiss Observer. The slots made from the longitudinal section of the samples were subjected to electrolytic etching in a 4% solution of nitric acid. The grain size was determined using the intersection method.

## 3. Results and Discussion

**Figure 1:**
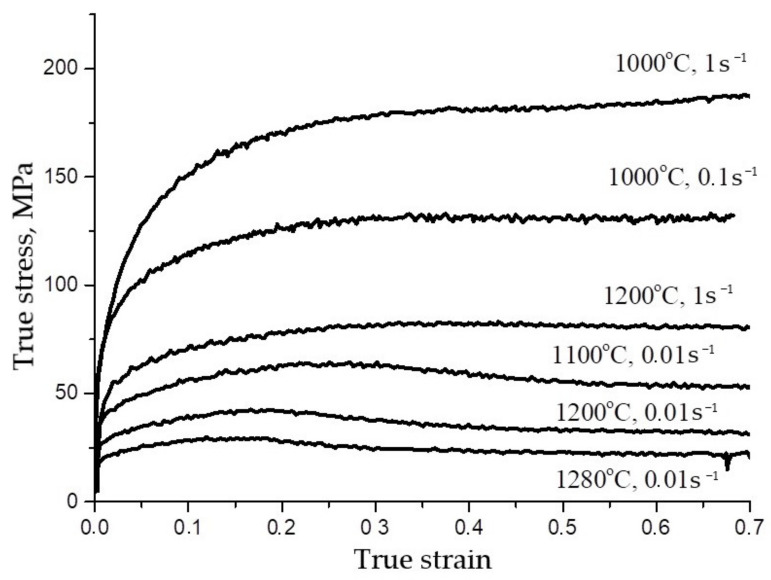
True stress-true strain cures of AISI 321 steel. The temperature and strain rate are shown in the figure.

Figure 1 represents some experimental stress-true strain curves for AISI 321 austenitic stainless steel. The flow stress decreased with the increase in deformation temperature and with the decrease in the deformation strain rate. This corresponds to the fact that the combined effect of temperature *T* and strain rate $\dot{\varepsilon }$ on the deformation behavior of metallic materials described by the Zener–Hollomon parameter as follows:

(1) $$Z=\dot{\varepsilon }\exp\left(\frac{Q}{RT}\right)$$

where Q is the deformation activation energy, R is the universal gas constant, and T is the absolute deformation temperature.

The flow stresses decrease with a decrease in the parameter *Z*, because an increase in temperature accelerates the thermally activated processes of dynamic recovery and dynamic recrystallization, and a decrease in the strain rate provides more time for their implementation. At relatively low temperatures and high strain rates (i.e., at high values of the *Z* parameter), a work-hardening stage was observed on the flow curves, after which the flow stress reached a constant (steady-state) level $\sigma_{s}$. The flow curves at a test temperature of 1000 °C with a strain rate of 0.1 s^−1^ and 1 s^−1^ have such a shape. The shape of flow curves shows that the hot deformation was accompanied by a dynamic recovery process—namely, the process of dynamic softening, which consists of the redistribution and annihilation of part of the dislocations. The work-hardening rate decreased monotonically with the increase in the strain and became zero when a balance was reached between the rate of generation and annihilation of dislocations, due to the dynamic recovery.

The stress curves obtained during tests with low values of the *Z* parameter (relatively high temperatures and low strain rates) have the form of curves with a maximum, indicating that hot deformation was accompanied by dynamic recrystallization. As shown in Figure 1, flow curves in the temperature range 1100–1280 °C with a strain rate of 0.01 s^−1^ have this shape. The formation of new recrystallized grains first led to a decrease in the strain work-hardening rate and the achievement of the peak value of the flow stress $\sigma_{p}$, and then to a drop in the flow stress, with their subsequent reaching a steady-state level $\sigma_{ss}$ corresponding to a completely recrystallized structure.

**Figure 2:**
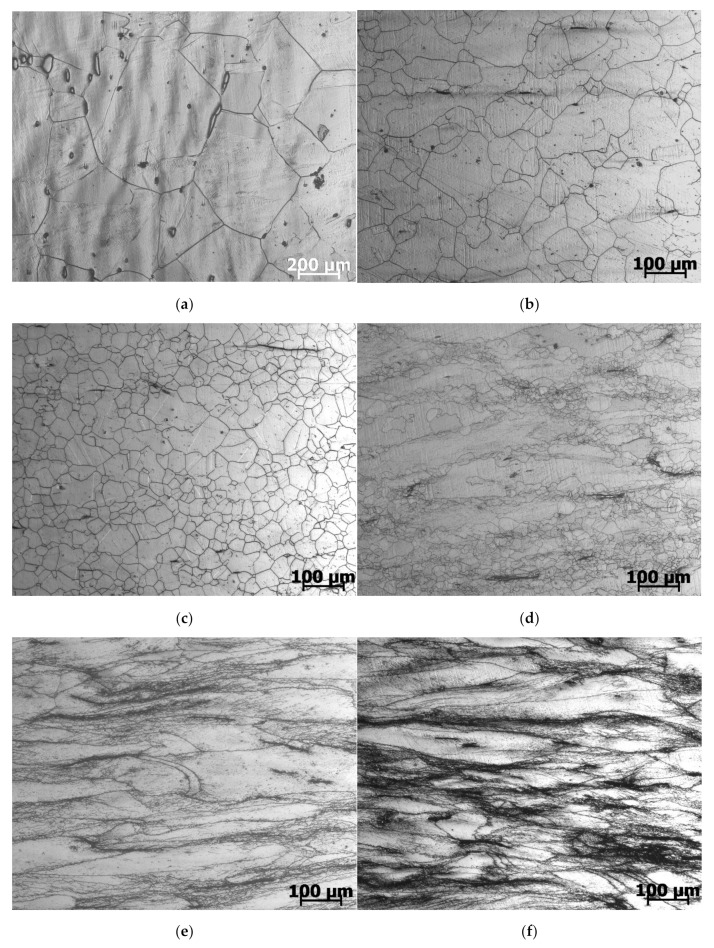
Microstructure of AISI 321 steel after heating to 1280 °C (**a**) and after hot deformation deformed at different temperatures and strain rates: (**b**) 1200 °C, 0.01 s^−1^; (**c**) 1200 °C, 1 s^−1^; (**d**) 1100 °C, 0.1 s^−1^; (**e**) 1000 °C, 0.01 s^−1^; (**f**) 1000 °C, 0.1 s^−1^.

The results of the microstructural studies are consistent with the shape of flow curves (Figure 2). During heating and 10 min holding at 1280 °C, a large austenitic grain with an average diameter of 300 µm was formed (Figure 2a). After deformation at temperatures of 1280 °C and 1200 °C, in accordance with the shape of stress-strain curves in Figure 1, the microstructure consisted of new dynamically recrystallized grains (Figure 2b,c). The size of recrystallized grains decreased with increasing strain rate and decreasing the deformation temperature, i.e., with an increase in the Zener–Hollomon parameter. Indeed, an increase in the strain rate accelerated the process of accumulation of dislocations and, accordingly, provided more sites for the nucleation of dynamically recrystallized grains and reduced the time for the growth of new grains. Lowering the deformation temperature reduces the mobility of grain boundaries and, thus, prevents grain growth [8].

The shape of the flow curve (with a stress peak) at a temperature of 1100 °C and a strain rate of 0.01 s^−1^ also indicates the passage of dynamic recrystallization. However, with an increase in the strain rate to 0.1 s^−1^, the time to complete the dynamic recrystallization was not enough, so some elongated non-recrystallized grains remained in the microstructure (Figure 2d).

**Figure 3:**
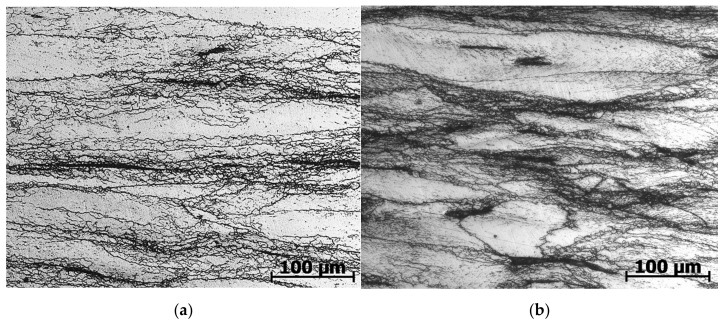
Microstructure of AISI 321 steel after hot deformation at 1000 °C: (**a**) with strain rate 0.01 s^−1^; (**b**) with strain rate 0.1 s^−1^.

Based on the flow curves at a temperature of 1000 °C, the flow stress monotonically increased to a steady level, i.e., there were no signs of dynamic recrystallization. The microstructure in Figure 2d,e with elongated non-recrystallized grains confirms this conclusion. Only along the boundaries of some grains and in the deformation bands, very small dynamic recrystallized grains were seen at higher magnification (Figure 3).

To find the relationship between the Zener–Hollomon parameter and peak or steady-state stresses, the empirical Sellars–Tegart expression [28] is widely used, which is a generalization of the power-law and exponential creep laws in the regions of low and high stresses, respectively,

(2) $$Z=\dot{\varepsilon }\exp\left(\frac{Q}{RT}\right)=A{\left[\sinh\left(\alpha \sigma_{m}\right)\right]}^{n}$$

where $\sinh\left(x\right)$ is the hyperbolic sine of the argument *x*; A, α, n are constants of the material to be determined. Here, the peak and/or steady flow stresses are denoted by a single symbol $\sigma_{m}$.

From Equation (2), it is derived that

(3) $$\sigma_{m}=\frac{1}{\alpha }\left(\mathrm{arcsinh}{\left(\frac{Z}{A}\right)}^{1/n}\right)$$

Unknown parameters in Equations (2) and (3) were found by regression analysis of experimental stresses $\sigma_{m}$ as follows:

(4) $$\begin{array}{cc} A=5.26\times 10^{16}\;s^{-1}; & \;\;\alpha =0.0074\;{\mathrm{MPa}}^{-1};\\ n=5.442; & \;\;Q=444.2\;\mathrm{kJ}/\mathrm{mol}. \end{array}$$

**Figure 4:**
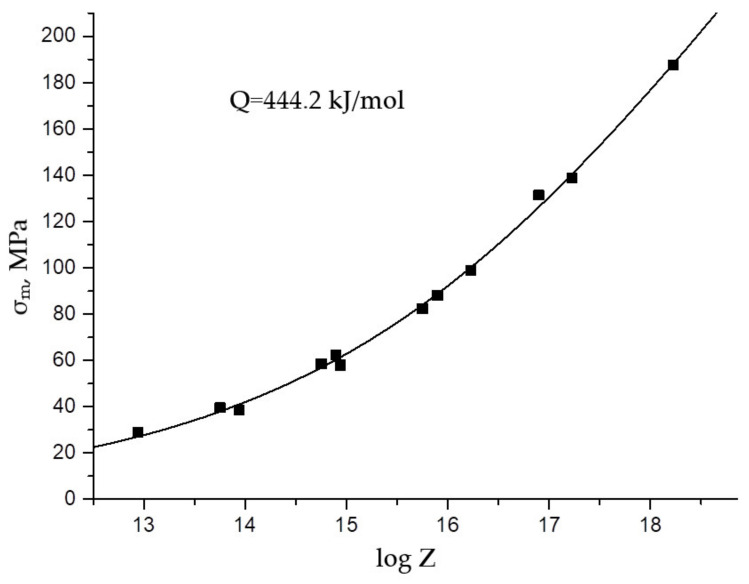
Experimental (point) and calculated (solid line) flow stress $\sigma_{m}$ as function of the Zener–Holloman parameter.

The flow stresses $\sigma_{m}$ calculated by Expression (3) using parameters (4) coincide with the experimental data with high accuracy (Figure 4).

A similar regression discrepancy can be obtained with slightly different sets of parameters *A*, *n*, and α. In this case, the activation energy *Q* changes by no more than 5 kJ/mol.

The exponent *n* (5.442) and the activation energy *Q* (444.2 kJ/mol) obtained by us lie within the ranges of values (4.5–6.1) and (433–465 kJ/mol), respectively, published earlier for AISI 321 steels with slightly different contents of carbon and titanium. However, all of the above estimates of the activation energy significantly exceed the value of the activation energy of self-diffusion of iron atoms in austenite (280 kJ/mol), which determines the rate of dislocation climb in the processes of dynamic recovery and the rate of migration of grain boundaries in the processes of dynamic recrystallization. One of the reasons for this discrepancy is the fact that Equations (2) and (3) do not take into account the temperature dependence of the elastic modulus of the material. Therefore, in [29,30], it is proposed in Expression (2) to use stresses normalized to the shear modulus $\left(\sigma_{m}/G\right)$ or Young’s modulus $\left(\sigma_{m}/E\right)$ instead of stresses $\sigma_{m}$. In this case, the activation energy drops to the value of the activation energy of self-diffusion, and the value of the exponent *n* close to 5, according to some authors, will indicate that the mechanism that controls hot deformation is glide and climb of dislocations [31,32]. However, at present, the precise expressions for the temperature dependence of elastic moduli are not known for all materials. Therefore, most authors continue to use the Sellars expression in the form (2), in this case calling the quantity *Q* the “apparent” activation energy.

After determining the apparent activation energy of investigated steel, it becomes possible to rank the studied deformation modes in ascending order of the Zener–Hollomon parameter *Z* (Table 2). Additionally, Table 2 shows experimental flow stress $\sigma_{m}$ and peak strain $\varepsilon_{p}$ (for deformation modes with dynamic recrystallization), the type of the forming microstructure (R—recrystallized, PR—partially recrystallized, NR—non-recrystallized), as well as the size *D* of dynamically recrystallized grains.

In the deformation modes accompanied by dynamic recrystallization, Expression (5) relates the peak strain $\varepsilon_{p}$ to the critical stain $\varepsilon_{c}$ for initiation of dynamic recrystallization [10] as follows:

(5) $$\varepsilon_{c}=k\varepsilon_{p}.$$

The coefficient *k* in Expression (5) for austenitic AISI 321 steel is 0.69 [21]. It is generally accepted that the peak strain is a power function of the Zener–Hollomon parameter.

(6) $$\varepsilon_{p}=aZ^{m}.$$

**Figure 5:**
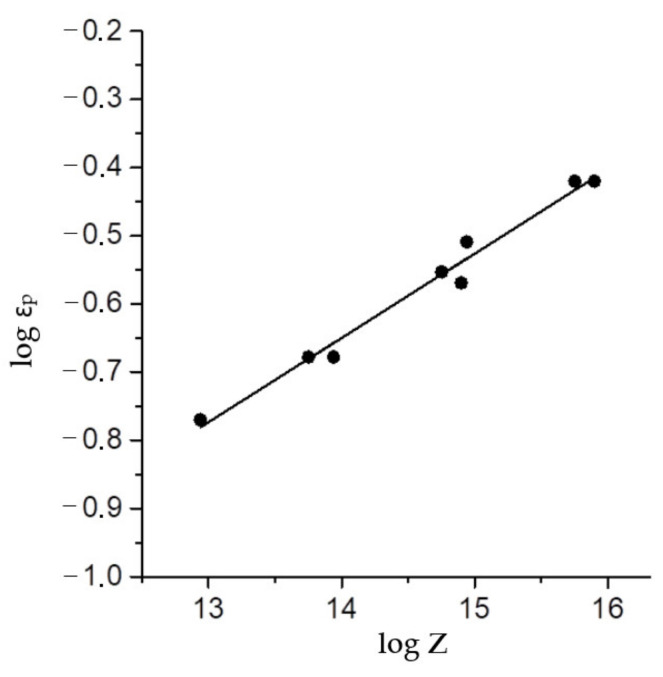
Peak strain as function of the Zener–Holloman parameter.

Indeed, the graph of the logarithm of peak strain versus the logarithm of the *Z* parameter has a linear form (Figure 5), from which we obtain the following equations by linear regression:

(7) $$\varepsilon_{p}=4.14\times 10^{-3}Z^{0.124},$$

(8) $$\varepsilon_{c}=0.69\varepsilon_{p}=2.86\times 10^{-3}Z^{0.124}.$$

The strain $\varepsilon_{f}$, at which the first cycle of dynamic recrystallization is completed, can be found as the strain at which the flow stress, after reaching the peak, drops to a steady-state level $\sigma_{ss}$.

Using experimental data $\varepsilon_{f}$, the following equation is found by regression analysis:

(9) $$\varepsilon_{f}=2.0\times 10^{-2}Z^{0.101}.$$

**Figure 6:**
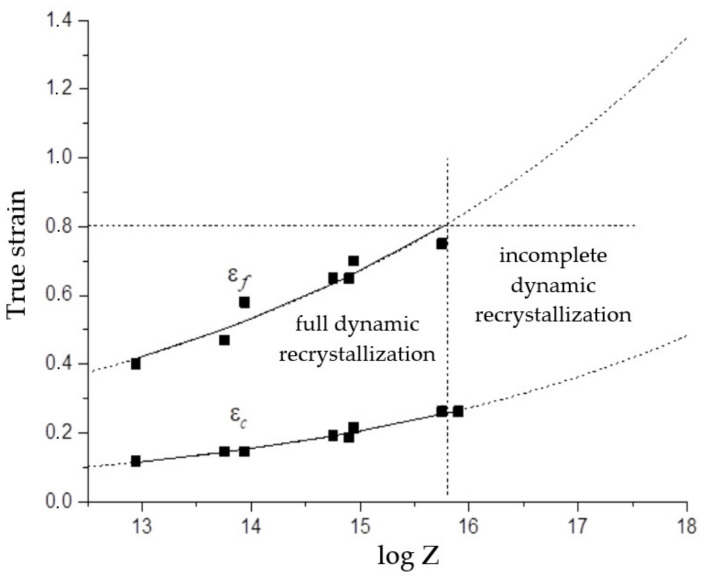
Critical strains $\varepsilon_{c}$ and $\varepsilon_{f}$ us function of the Zener–Holloman parameter.

The results of the calculation of critical strains $\varepsilon_{c}$ and $\varepsilon_{f}$ by Expressions (8) and (9) as well as the experimental values are shown in Figure 6.

The horizontal dotted line in Figure 6 shows the true deformation (0.8), up to which compression tests were carried out. As can be seen, in all deformation modes with parameters log *Z* ≤ 15.75, the deformation $\varepsilon_{f}$ required to complete the first cycle of dynamic recrystallization was below 0.8. Consequently, the dynamic recrystallization during the tests was complete. This conclusion was confirmed by the results of microstructural studies (Figure 2b,c). The vertical dotted line in Figure 5 corresponds to the value log *Z* = 15.75 (*Z* = 5.6 × 10^15^), which limits the area of full dynamic recrystallization in our investigation.

According to the graphs of Equations (8) and (9) extrapolated to the high *Z* region (dotted curves in Figure 5), with a further increase in the log *Z* parameter, a monotonous decrease in the portion of dynamically recrystallized grains in the total volume of the material should occur. However, as follows from the microstructural data, even with a small increase in *Z* to the value log *Z* = 15.9 (temperature of 1100 °C, strain rate of 0.1 s^−1^), the portion of dynamically recrystallized grains decreased sharply (Figure 2d). With a subsequent small increase in the parameter *Z* to the value log *Z* = 16.23 (temperature of 1000 °C, strain rate of 0.01 s^−1^), only narrow bands of very small recrystallized grains along the boundaries of large elongated grains are visible in microstructure photographs (Figure 3a). From the presented results, it can be concluded that, in the studied steel in deformation modes with parameters log *Z* > 15.75, instead of a monotonous decrease in the fraction of dynamically recrystallized grains, a sharp suppression of dynamic recrystallization occurred.

It should be noted that in AISI 321 steel with a lower content of carbon (0.04%) and titanium (0.32%), deformation at a temperature of 1000 °C with strain rates in the range of 0.01–1 s^−1^ (i.e., in the log *Z* range of 16.23 to 18.23) was accompanied by complete dynamic recrystallization [23]. This circumstance suggests that the reason for the suppression of dynamic recrystallization in our AISI 321 steel with a high content of carbon (0.07%) and titanium (0.50%) can be dispersed particles of titanium carbonitrides Ti(C, N) that precipitate during hot deformation and suppress the growth of new grains.

This circumstance suggests that the reason for the suppression of dynamic recrystallization in our AISI 321 steel with a high content of carbon (0.07%) and titanium (0.50%) can be dispersed particles of titanium carbonitrides Ti(C, N), which precipitate before and/or in the process of hot deformation and suppress the growth of new grains.

As shown in [33], the trace amount of nitrogen dissolved during solution treatment significantly increases the chemical driving force for Ti(C,N) nucleation and therefore accelerates the rate of precipitation. Accordingly, the precipitation kinetics in our steel with 0.014% N was significantly faster than in the steel studied in [22,23,24,25] and that containing only 0.001% N [25]. Indeed, dispersed Ti(C, N) precipitates were detected in a similar study on steel via transmission electron microscopy [30]. Another factor delaying the processes of dynamic recrystallization of the steel under study is a large initial grain of austenite formed at high initial preheating of the samples to 1280 °C and 10 min exposure at this temperature (in [22,23,24,25], the heating temperature before deformation was 1200 °C). The boundaries of the initial austenite grains serve as the nucleation sites of dynamically recrystallized grains. Therefore, with an increase in the size of the initial grains, accompanied by a decrease in the area of their boundaries, there is an increase in the critical deformation required for the start of dynamic recrystallization [19].

Elucidation of the true reasons for the suppression of dynamic recrystallization of the steel under study in deformation modes with the parameter log *Z* > 15.75 requires further research. Nevertheless, it can be argued that, for guaranteed refinement of the grain structure of AISI 321 steel due to dynamic recrystallization, the temperature of hot deformation should not be lower than 1100 °C.

**Figure 7:**
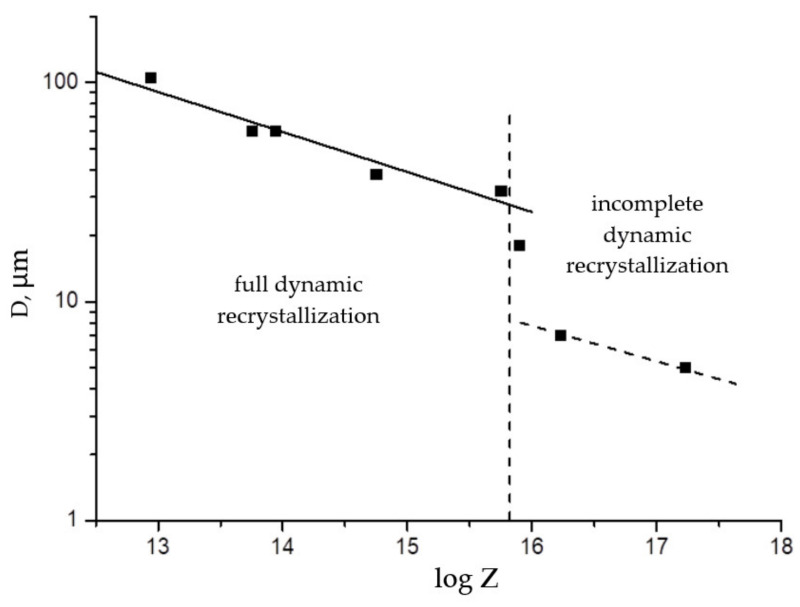
Recrystallized grain size as a function of the Zener–Hollomon parameter.

To select rational industrial modes of hot deformation, it is necessary to obtain an analytical expression for the size *D* of dynamically recrystallized grains as a function of the Zener–Hollomon parameter. For this purpose, the graph of the logarithm of experimental grain sizes versus log *Z* was plotted (black dots in Figure 7). In the region of full dynamic recrystallization, i.e., if log *Z* < 15.75, the graph is linear. Thus, we define an expression for the size of dynamically recrystallized austenite grains in the form of a power function of the Zener–Hollomon parameter as follows:

(10) $$D=2.1\times 10^{4}Z^{-0.183}\;\;(\mu m)$$

In the case of the formation of a microstructure with incomplete (suppressed) dynamic recrystallization, the sizes of small grains formed along the boundaries of large deformed grains are determined with a significant error; however, it can be argued that their corresponding points lie significantly below the regression line. Expression (10) is valid in the region of full dynamic recrystallization and makes it possible to determine the temperature range and the rate of hot deformation, which ensures obtaining grains of the required size in a completely recrystallized microstructure.

## 4. Conclusions

The hot deformation behavior of austenitic AISI 321 steel with a relatively high content of carbon (0.07 wt.%) and titanium (0.50 wt.%) was studied in the temperature range of 1000–1280 °C and strain rates in the range of 0.01–1 s^−1^. The main results are as follows:

(1) The apparent activation energy of hot deformation in the Zener–Hollomon parameter *Z* was 444.2 kJ/mol. The flow stresses decreased with a decrease in the *Z* parameter, i.e., with an increase in temperature and a decrease in the strain rate.
(2) The hot deformations were accompanied by full dynamic recrystallization if log *Z* < 15.65 or *Z* < 5.6 × 10^15^. The size of recrystallized grains is a power function of the Zener–Hollomon parameter and decreases with increasing temperature and increasing strain rate.
(3) In deformation modes with parameters log *Z* > 15.65, a sharp suppression of dynamic recrystallization was observed. The reason for the suppression of dynamic recrystallization in AISI 321 steel can be dispersed particles of titanium carbonitrides Ti(C, N), which precipitate before and/or during hot deformation and hinder the growth of new grains.
(4) The analytical expressions obtained in this research for flow stresses, the critical deformation of the onset of dynamic recrystallization, and the size of recrystallized grains are suitable for use in finite element modeling of hot deformation processes in the steel under study.

## Figures and Tables

**Table 1:** Chemical composition (wt.%) of the AISI 321 steel.

C	Si	Mn	Cr	Ni	S	P	Mo	Cu	Ti	N
0.07	0.30	1.33	17.7	10.3	0.005	0.025	0.19	0.19	0.50	0.014

**Table 2:** Deformation modes and their corresponding characteristics.

*T* (°C)	*ε′* (s^−1^)	log *Z*	$\sigma_{m}\;(\mathrm{MPa})$	$\varepsilon_{p}$	Type of Microstructure	*D* (μm)
1280	0.01	12.94	29	0.17	R	105
1200	0.01	13.75	39	0.21	R	60
1280	0.1	13.94	38	0.21	R	60
1200	0.1	14.75	59	0.28	R	38
1100	0.01	14.90	62	0.27	R	-
1280	1	14.94	58	0.31	R	-
1200	1	15.75	82	0.38	R	32
1100	0.1	15.90	88	0.38	PR	-
1000	0.01	16.23	99	-	NR	-
1100	1	16.90	131	-	NR	-
1000	0.1	17.23	139	-	NR	-
1000	1	18.23	188	-	NR	-

## Data Availability

Data sharing is not applicable.
